# Adaptation of *Coping Together* - a self-directed coping skills intervention for patients and caregivers in an outpatient hematopoietic stem cell transplantation setting: a study protocol

**DOI:** 10.1186/s12913-018-3483-1

**Published:** 2018-08-29

**Authors:** Tammy Son, Sylvie Lambert, Ann Jakubowski, Barbara DiCicco-Bloom, Carmen G. Loiselle

**Affiliations:** 1 Department of Survivorship and Supportive Care, MSKCC, 1275 York Avenue, New York, NY 10065 USA; 2 Ingram School of Nursing, McGill University, Montreal, H3A 2A7 Canada; 3 Adult Bone Marrow Transplantation Service, MSKCC, 1275 York Avenue, New York, NY 10065 USA; 4 Department of Nursing, City University of New York, 365 Fifth Avenue, New York, NY 10016 USA; 5 Department of Oncology, McGill University, Montreal, Canada; 6 Segal Cancer Centre, Jewish General Hospital, Montreal, Canada

**Keywords:** Hematopoietic stem cell transplantation, Dyads, Caregivers, Coping, Recovery phase, Self-directed intervention, Intervention adaptation and refinement, Center for Disease Control and Prevention’s map of adaptation, *Coping together*

## Abstract

**Background:**

Despite numerous reports of significant distress and burden for hematopoietic stem cell transplantation (HSCT) patients and caregivers (CGs), HSCT-specific coping interventions remain rare. The few in use lack specificity and are often not easily accessible or cost-effective. Whereas the development of new interventions is resource-intensive, theory-informed adaptation of existing evidence-based interventions is promising. To date, no HSCT-specific intervention has relied on a formal adaptation approach.

**Methods:**

Using the Center for Disease Control and Prevention’s Map of Adaptation, this two-phase qualitative descriptive study seeks to understand the perceptions of HSCT patients, CGs, individually, and in dyads, and clinicians about *Coping Together* (CT) for the preliminary adaptation (Phase 1), and then explores perceptions of the modified intervention in additional mixed sample (Phase 2). Six to ten participants including outpatients, CGs and dyads and five to seven HSCT clinician participants will be recruited for Phase 1. For Phase 2, 14 to 16 participants including outpatients, CGs and dyads will be recruited. Individual and dyadic semi-structured interviews will take place between 100 and 130 days post-HSCT. Verbatim transcripts will be analyzed using content analysis.

**Discussion:**

It is paramount to have HSCT-specific supportive interventions that address patients’ and CGs’ multidimensional and complex needs. The timely involvement of key stakeholders throughout the adaptation process is likely to optimize the relevance and uptake of such tailored intervention.

**Trial registration:**

This study is registered on October 6, 2016 in ClinicalTrials.gov at (identifier number NCT02928185).

## Background

In 2015, nearly 20,000 autologous (self-donor) and allogeneic (related or unrelated donor) hematopoietic stem cell transplantations (HSCTs) were performed in the US with expectations of a continued upward trend [1]. HSCT for hematologic malignancies, such as leukemia, lymphoma, multiple myeloma, myelodysplastic syndrome and aplastic anemia, is a potentially curative procedure, and in cases of refractory or advanced disease, remains the last hope [2]. Traditionally, HSCTs have been inpatient procedures due to intensive treatment regimens and a complex recovery process. However, feasibility, cost containment, and patients’ needs and quality of life issues have mandated a shift to outpatient settings [3–5]. This shift means more intensive involvement of caregivers (CGs).

With an increasing number and complexity of role demands, CGs are challenged to perform their role while balancing other demands with limited preparation and support [6]. CGs, therefore, are prone to illness-related distress, unmet needs and coping difficulties that often exceed that of patients [7–9]. Frequently, the necessity of balancing competing demands leads to levels of distress (anxiety, depression) that are comparable to psychiatric inpatients, thus compromising the caregiving role [10, 11]. In addition, concerns prevail about the perseverance of HSCT CGs to sustain their vital role over a prolonged course and how to support them most effectively.

Acknowledging the shared cancer experience of patients and CGs, clinicians and researchers alike have begun exploring intervention development from a dyadic perspective [12]. Conceptualization of cancer as a disease affecting more than the patient emanates from the notion that “illness is something that happens to the couple and that the focus on patient and partner separately may not be as beneficial from a theoretical and clinical perspective as a focus on the relationship” ([13], p., 2542). Evidence suggests that positive dyadic coping can enhance patients’ and partners’ psychosocial adjustment [14, 15]. Hence, coping strategies adopted by patients facilitate their own well-being as well as their partners’ and vice versa [16, 17].

Thus, couple-based interventions (CBIs) that involve dyads, namely patients and their spouse/partners, are promising in terms of diminishing psychological distress associated with a stressful chronic illness such as cancer [18]. However, CBIs have rarely been integrated into routine care due to several barriers including patients being too busy to participate, limited intervention access, the lack of tailored interventions and costs related to reliance on trained professionals [14, 15, 19].

Moreover, limited intervention access is evident in the HSCT population. In a randomized control trial (RCT) with 148 partners of allogeneic patients, Laudenslager et al., reported positive outcomes with a modified version of a psychosocial intervention on psychological and physiological distress, anxiety and depression during the first 3 months post-HSCT [20]. CGs in the intervention group demonstrated a downward trend in psychological distress, anxiety and depression over 3 months with effect sizes ranging from 0.39 to 0.66 [20]. Despite the favorable impact of the intervention, researchers’ costs ranging from $300 to $350 per participant hindered sustainability. In another HSCT invention study, Bevans et al., demonstrated the feasibility of an individualized problem-solving education intervention during the first 4 weeks after discharge home [21]. However, in addition to excessive researcher costs, they also cited challenges in coordinating schedules between the researcher and the participants, and time constraints of CGs who need to balance multiple roles [21]. Furthermore, none of the cited studies detailed the adaptation process for their respective intervention.

Recently, self-directed (unguided or without a researcher/clinician involved in delivery; also known as self-administered, self-help) interventions have been suggested as a cost-sparing, flexible, accessible and promising delivery modality for cancer dyads [22, 23]. The self-directed delivery modality allows users to self-determine what (content), when (time), where (location) and how (individually or jointly) to use the intervention booklets. Therefore, self-directed delivery formats can provide HSCT patients and CGs the benefits of flexibility in timing and diminish potential information overload [24].

### The *Coping Together* (CT) intervention

CT is an evidence-informed, self-directed individual and dyadic coping skills intervention that has been developed in Australia to enhance adaptive coping strategies for heterogeneous cancer patients and partners confronting physical and psychosocial challenges [25]. Preliminary data from a pilot study demonstrate positive trends for CT patients in decreasing distress (intrusion and avoidance) and appraising cancer as significantly less challenging, whereas, for partners, CT was related to lower caregiver stress, reduced financial strain and improved illness-related appraisal [19]. In an ongoing RCT, the efficacy of CT is examined in prostate, breast, colorectal and early stage melanoma patients and their partners as a multimedia intervention comprises of six booklets, a DVD of key content, a relaxation CD and a website [23]. The series of six CT booklets addresses: (1) symptom and side effect management; (2) developing quality relationships with healthcare providers; (3) soliciting additional support; (4) improving cancer-related communication pathways; (5) contending with emotions and worries; and (6) coping with treatment-related decisions. Each booklet presents cognitive-behavioral therapy-based exercises to facilitate tailored coping strategies, suggestions for contending with potential stress-inducing situations, anecdotal accounts, and suggestions from clinicians [23].

### Adaptation of the *Coping-Together* booklets

Adaptation is the process of modifying an evidence-based intervention to minimize disparities between its characteristics and those of the new target group [26]. Intervention adaptation can be minor (e.g., changing the name) or substantial (e.g., revising its clinical or cultural evidence). The adaptation process of the CT booklets will retain the core components that produce its intended outcomes, such as the enhancement of adaptive coping skills and greater confidence in the use of these skills, individual and dyadic involvement, and adoption of new pragmatic coping strategies [27]. Whereas key characteristics, important attributes of the intervention, will be adapted according to clinical judgment and recommendations of participating HSCT patients, CGs, dyads and clinicians [27]. Key characteristics will include the multimedia format, structure (e.g., length), content (e.g., HSCT-related information, coping skill strategies), information presentation (e.g., font size, text boxes, visuals), cognitive-therapy based exercises, and supportive narratives. The booklet concerning treatment-related decisions will be excluded, as this is irrelevant during the post-HSCT recovery phases.

### CDC’s Map of Adaptation guidelines

The CDC’s Map of Adaptation is a systematic approach that emphasizes implementer and clinician involvement to ensure adaptation and tailoring to the target group [27]. The Map of Adaptation also allows feedback loops to ensure adaptation is tailored to the new target population and flexibility in step sequence (allowance for non-sequential, simultaneous step sequence) [27]. For this study, the first three steps will be completed, which are instrumental for informing the acceptability of the intervention; intervention refinement and laying the foundation for subsequent pilot intervention studies [28–30]. The Steps of the Map of Adaptation guidelines are detailed below.

Step 1 called “*Assess*” involves three parts. Part 1 comprises a literature review of the target population focusing on their needs, socio-demographics and recent trends (treatment-related, health-seeking behaviours), as well as determining the suitability of the intervention for the target population and any barriers potentially limiting access to the intervention. Part 1 can include interviews of key stakeholders. Part 2 entails a literature review of potential interventions that can meet the target population’s needs. In Part 3, the organizational capacity to adapt and implement the intervention is reviewed by considering its philosophy (mission, values), experiences (with the target population, implementing interventions) and resources (staff, supplies, space, funding).

Step 2 called “*Select*” evaluates the recommendations from Step 1, and then finalizes the decision regarding which intervention will be adapted. In most cases, there are discrepancies among the target population, intervention and organization, thereby directing the need for adaptation that can be minor such as the revision of a term used, or substantial such as revising the intervention’s cultural relevance for the new setting.

Step 3 called “*Prepare*” involves three parts. In Part 1, an adapted intervention based on the stakeholders’ recommendations is generated. In Part 2, participants’ experiences with the adapted intervention are pre-tested for further refinement to ensure tailoring and cultural appropriateness of materials. Part 2 focuses on content, language translation, intervention renaming, incorporating local proverbs and anecdotes, as well as identifying potential implementation issues, such as delivery modality, literacy level, layout, and clarity of instructions. In Part 3, the organization is prepared to ensure successful implementation by recruiting and training additional staff, obtaining (different) space, assembling materials, securing more funding, and strengthening partnerships and collaborations.

**Fig. 1:**
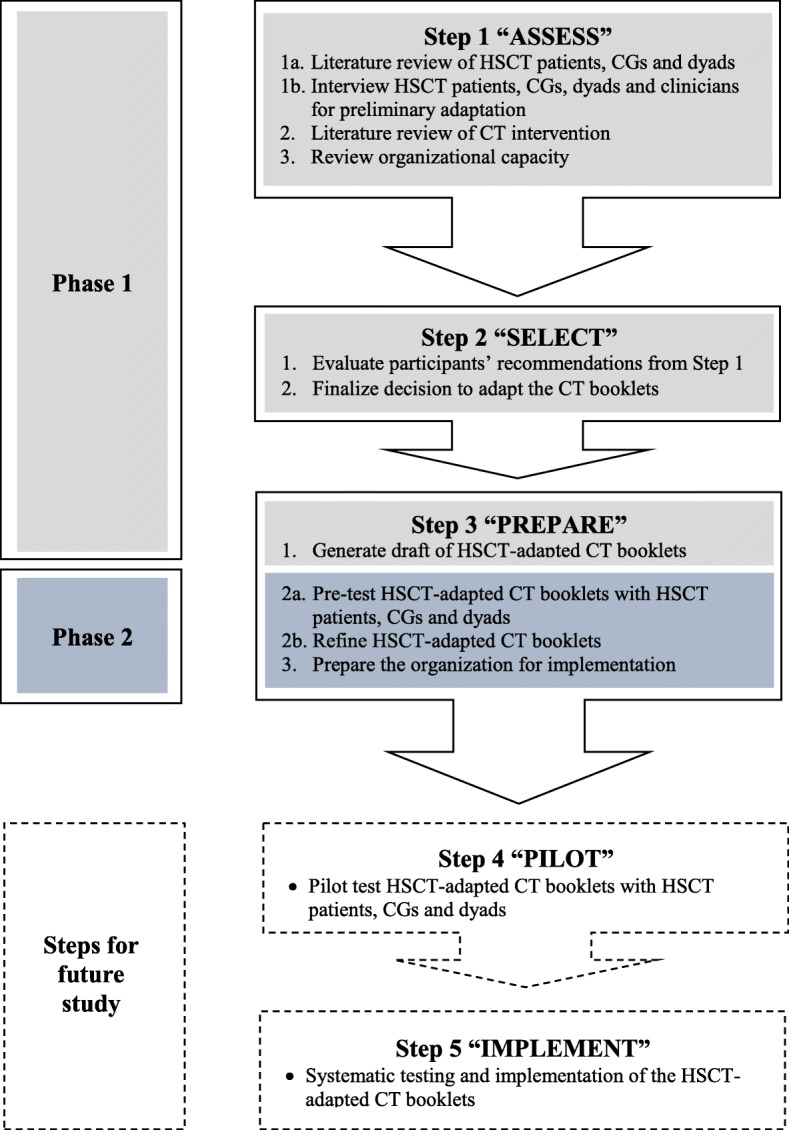
Summary of the CDC’s Map of Adaptation Guidelines

Step 4 called “*Pilot*” and Step 5 called “*Implement,*” which involve testing and implementing the adapted intervention, respectively, will not be discussed in detail as they are beyond the scope of this study. Furthermore, Phase 1 of this study will include Steps 1, 2 and 3, Part 1 to generate the HSCT-adapted CT intervention (hereafter cited as HSCT CT), and Phase 2 will include Step 3, Parts 2 and 3 to refine the HSCT CT booklets (Fig. 1).

### Study aims and research questions

This two-phase descriptive qualitative study will expand the current literature by using the CDC’s Map of Adaptation guidelines to systematically adapt the CT booklets for the HSCT context. Phase 1 will gather the perceptions of participating HSCT patients, CGs, dyads and clinicians about the unmodified CT booklets for preliminary adaptation. Phase 2 will explore the experiences of an additional sample of HSCT patients, CGs and dyads to further refine the HSCT-adapted CT booklets.

Specifically, this study poses three research questions:

- Guided by the CDC’s Map of Adaptation, what are the perceptions of HSCT patients, CGs, dyads and clinicians about the unmodified CT booklets in the early post-HSCT period?;
- What needs to be understood about the participants’ perceptions about the unmodified CT booklets?; and
- What are the participants’ perceptions/experiences with the HSCT-adapted CT booklets during the first 100 days post-HSCT?

## Methods/design

### Study design and setting

**Fig. 2:**
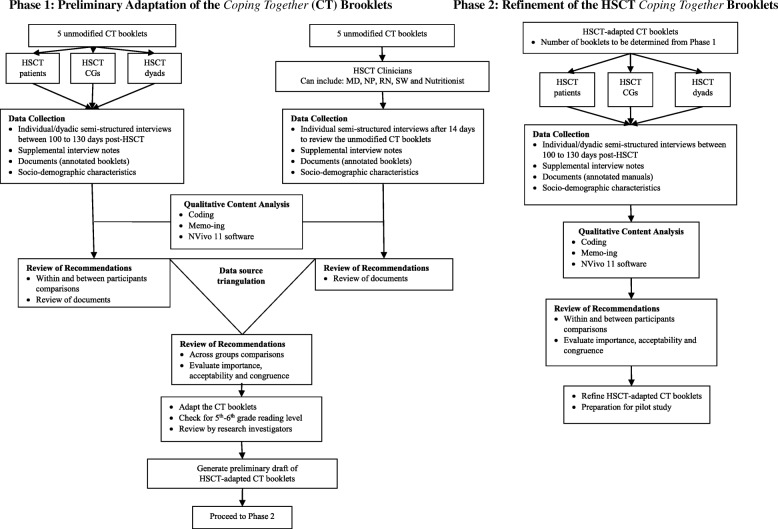
Study Schemas

The study employs a qualitative descriptive design. Participants will include patients in the first 100 days after being treated for an outpatient HSCT at Memorial Sloan-Kettering Cancer Center (MSKCC) in New York City, USA. The study design is illustrated in Fig. 2. For this study, CG will be defined as spouse, partner, family member, friend, colleague, or neighbor who provides uncompensated medical (e.g., aseptic central line care) and custodial (e.g., preparing low microbial diet) care who might, but does not necessarily lives with the patient [31–33]. A dyad will be defined as a combination of a patient and their designated CG. Patients will be included if they are admitted to the Adult Bone Marrow Transplantation (BMT) service for an outpatient HSCT for a hematologic malignancy. Other inclusion criteria for patients and CGs will be age greater than 18 years; ability to read, speak and understand English; and physically and cognitively able to participate in the study. CGs will be excluded if they are unable to fulfill caregiver responsibilities for more than 50% of the time (> 50 days). Dyads will be included if patients and CGs mutually agree to participate in the study.

### Phase 1: Preliminary adaptation of the CT booklets

#### Recruitment of Phase 1 HSCT patients, CGs and dyads

A convenience sample of six to ten patients, CGs and dyads will be recruited. Data saturation will not be attained since the objective is to obtain diverse perspectives of key stakeholders with the unmodified CT booklets for the preliminary adaptation per the CDC’s Map of Adaptation guidelines.

#### Procedures for recruitment of Phase 1 HSCT patients, CGs and dyads

The first author will screen potential participants who are listed on the weekly BMT admission schedule and then, will present the study at an introductory meeting during which questions will be answered. Amenable participants will sign the consent form and complete a socio-demographic questionnaire. Each participant will be provided a copy of the signed consent form.

Within 1 month prior an outpatient HSCT, the first author will deliver an intervention pack containing the following: (a) five unmodified CT booklets (one set for HSCT patients and CGs and two sets for HSCT dyads); (2) instructions on how to use the booklets; (3) instructions to review the content and mark up the copies ad lib with handwritten comments and recommendations; and (4) a reminder to bring the annotated CT booklets to the interview to facilitate the discussion. Between 50 and 75 days post-HSCT, the first author will remind HSCT patients, CGs and dyads to review the CT booklets. This communication will be a prompt only and not for therapeutic counseling. Between 90 to 100 days post-HSCT, the first author will contact participants to arrange individual or dyadic interviews that will take place between 100 and 130 days post-HSCT.

#### Data collection of Phase 1 HSCT patients, CGs and dyads

Qualitative data will be collected from semi-structured interviews, supplemental interview notes, and documents (annotated booklets). All participants will also complete a socio-demographic questionnaire.

Between 100 and 130 days post-HSCT, the first author will conduct semi-structured, face-to-face or telephone, individual and dyadic interviews to describe the participants’ perceptions about the content, structure and process of the unmodified CT booklets and recommendations for the preliminary adaptation. The interview format – face-to-face versus telephone -- will be based on each participants’ convenience to reduce study burden.

Following an interview guide, participants will be queried with open-ended questions designed to address the research focus (Table 1). Questions will be intentionally broad and open-ended to encourage participants to share their own perspectives. Flexibility in the question sequence will be allowed to facilitate conversation flow. Interview techniques will include (1) probes to increase data richness and depth of responses; (2) interpreting questions based on cues from something said, alluded to or to revisit an issue superficially discussed; (3) explicating the implied (repeating what has been said in the form of a question) to facilitate elaboration; and (4) silence to allow for reflection and formulation of responses [34]. Probes will be used to ensure feedback is obtained from each dyadic member. Interviews will be audio-recorded, transcribed verbatim by a professional transcription service and supplemented by interview notes. Estimated duration of interviews will be 60 min. The annotated booklets will be collected and reviewed to corroborate information from the interviews and for data source triangulation.

**Table 1:** Summary of Phase 1 Semi-Structured Interview Questions

Participants	Interview Questions
HSCT Patients or CGs	• Could you please tell me what it has been like during the first 100 days after HSCT? • How did you use the booklets during the first 100 days after HSCT? • Approximately how much of each booklet (percent or number of pages) would you estimate that you reviewed? If you didn’t review the booklets, could you please tell me the reason(s)? • What was helpful/least helpful? • How did you find using these booklets on your own? Would it have been better to use them together? • What did you think about the • Layout? • Appearance? • Information? • What would you keep/remove? • What other recommendations would you make?
HSCT Dyads	• Could you please tell me about your experiences as a couple within the first 100 days post-HSCT? • Can you describe how you cope with difficult situations as a couple? • Approximately how much of each booklet (percent or number of pages) would you estimate that you reviewed? If you didn’t review the booklets, could you please tell me the reason(s)? • How much of the time did you use the booklets together? And as individuals? • What content will be helpful to you as a couple? • What content will be least helpful as a couple? • What changes do you recommend?
HSCT Clinicians	• Could you please tell me what you think about the content of the CT booklets for HSCT patients, CGs and dyads? • Could you give me examples of what you think will be helpful/least helpful? Why? • What do you think are the advantages/disadvantages of the self-directed format for HSCT dyads? • What information do you think is missing? • What changes would you recommend?

#### Data analysis Phase 1 HSCT patients, CGs and dyads

Qualitative data will be analyzed using inductive Qualitative Content Analysis (QCA) and NVivo® (QSR International, Doncaster, Australia) to obtain a comprehensive, descriptive summary of opinions, perceptions and experiences in the words of the participants [35]. QCA will identify themes for recommendations for adapting the CT booklets. Data from the interviews will be analyzed separately, and then compared for discerning differences in the participants’ perceptions as well as areas of consensus on changes to be integrated into the booklets. Intra-dyadic comparisons will be conducted to discern similarities and differences between joint and individual recommendations and perceptions. Dyadic data of each member will also be compared with recommendations from individual HSCT patients and CGs, respectively. Data collection and analysis will be conducted concurrently and sequentially in the following three phases:

##### Preparation

Data will be prepared by establishing the unit of analysis and meaning analytic units. The unit of analysis will be the text of the transcripts as well as observations recorded after interviews (supplemental notes), whereas meaning units will be words and phrases that are similar or have the same fundamental meaning, and provide insights in answering the research questions [35].

##### Immersion

Immersion in the data will proceed by reading the transcripts multiple times to make sense of the data and to gain an understanding of the whole of the data in the words of the participants. Open coding will be completed from the word-to-word review of the manuscripts. After reading the first two to three transcripts, preliminary codes that will be recorded will be searched for in the remaining transcripts to confirm data-driven codes (in vivo). Then, the transcripts will be uploaded into NVivo® to organize codes that capture key thoughts and concepts, link data to memos and generate visual models of the data. Memos will be recorded throughout the analysis, wherein the first author will reflect on the data; record initial thoughts and reactions to the data, describe codes, categories, comparisons and interpretations; brainstorm new ideas; and present new questions to understand who, what, why, and when the emerging themes had meaning.

##### Abstraction

Data will be abstracted by formulating a general description of the HSCT participants’ perceptions of the booklets and areas in need of adaptation. Meaning units will be condensed into codes, which are word(s) from the transcripts that will be organized into categories and sub-categories. Each code will be constantly compared to the other codes to discern emerging similarities, discrepancies and general patterns. Coded data will also be compared within similar groups. Categories will be grouped by collapsing into other groups based on similarities and differences. After repeated coding, relevant concepts and content will be clustered into emergent themes that link the underlying meanings of categories together.

Socio-demographic characteristics will be summarized with descriptive statistics (frequencies, percentages, means, standard deviations) using IBM SPSS Statistics, Version 22. These will be computed separately for patients and CGs.

#### Recruitment of Phase 1 HSCT clinicians

A convenience sample of five to seven multidisciplinary HSCT clinicians will be recruited. Clinicians who will be included must be members of the HSCT patient care team with at least one-year experience.

#### Procedures for recruitment of Phase 1 HSCT clinicians

The first author will email potential clinicians a study overview, anticipated time commitment, and an invitation to participate. The first author will deliver the verbal consent confirming voluntary participation as approved by the Chairman of the Institutional Review Board (IRB)/Privacy Board at MSKCC. After obtaining verbal consents, the first author will provide five unmodified CT booklets with instructions to review the contents and mark up the booklets ad lib with handwritten comments.

#### Data collection of Phase 1 HSCT clinicians

After at least 14 days for booklet review, the first author will arrange a face-to-face or telephone, semi-structured interview for each HSCT clinician. Questions from the interview guide are detailed in Table 1. Interview procedures and techniques, recording supplemental interview notes, and collection of annotated booklets and descriptive data will be the same as described above. Estimated duration of interviews will be 60 min.

#### Data analysis Phase 1 HSCT clinicians

Qualitative data and socio-demographic characteristics will be analyzed using the same data analysis procedures as described above.

#### Adaptation process of CT booklets

The recommendations of all participants will be systematically evaluated based on (1) perceived importance (e.g., related to the improvement to CT’s effectiveness); (2) acceptability (e.g., the burden level for the HSCT patients, CGs, dyads, clinicians, and the study site); and (3) congruence (e.g., the compatibility with CT’s core components) [36]. The developer will review a list of recommendations to ensure the integrity of the CT booklets is maintained. If discrepancies arise among researchers, the recommendations will be incorporated into the HSCT-adapted CT booklets so that Phase 2 participants can review and comment on them. Content will be checked for fifth to sixth grade reading level using an online readability program.

### Phase 2: Refinement of the HSCT CT booklets

Phase 2 will explore the experiences of an additional sample of HSCT patients, CGs and dyads to refine the HSCT-adapted CT booklets to ensure they will be adapted to address their needs. Phase 2 will focus on Steps 3, parts 2 and 3 of the CDC’s Map of Adaptation (Fig. 1).

#### Recruitment of Phase 2

An additional convenience sample of HSCT patients, CGs and dyads will be recruited to gain further insights into their experiences with the HSCT-adapted CT booklets and refine them as necessary. The sample size is estimated to be 14 to 16 participants, but will be determined by data saturation. Data saturation is when no new insights and themes emerge (data redundancy) indicating that sufficient data has been acquired to account for all facets of the phenomenon [37, 38]. Sampling strategies and eligibility criteria will follow the same procedures as described in Phase 1 HSCT Patients, CGs and Dyads.

#### Procedures for recruitment of Phase 2

The same procedures for recruitment will be followed as described for Phase 1 HSCT Patients, CGs and Dyads.

#### Data collection of Phase 2

The same procedures for data collection, namely individual and dyadic interviews (Table 2), supplemental interview notes, annotated booklets and socio-demographic characteristics will be followed as described for Phase 1 HSCT Patients, CGs and Dyads.

**Table 2:** Summary of Phase 2 Semi-Structured Interview Questions

Participants	Interview Questions
HSCT Patients or CGs	• How did you find the information that was in the booklets? • Approximately how much of each booklet (percent or number of pages) would you estimate that you reviewed? If you didn’t review the booklets, could you please tell me the reason(s)? • Could you rank the booklets in order of usefulness? • Could you tell why booklet that (referring to the number one ranked booklet) was the most helpful? • Could you tell me why the booklet (referring to the least ranked booklet) was the least helpful? Should it be removed? • Could you tell me what other ways do you like to get information about your health? • What do you think needs to be changed? What would you remove/keep? • Is there any information that you think is missing?
HSCT Dyads	• How did you find the information that was in the booklets? • Approximately how much of each booklet (percent or number of pages) would you estimate that you reviewed? If you didn’t review the booklets, could you please tell me the reason(s)? • Could you rank the booklets in order of usefulness? Why was the booklet ranked the most useful? Why was the booklet ranked least useful? Should this booklet be removed? • How often did you use the booklets together? And as individuals? • In what ways did the booklets help you deal with HSCT-related issues together? • Could you tell me what other ways do you like to get information about your health? • What do you think needs to be changed? What would you remove/keep? • Is there any information that you think is missing?

#### Refinement process of HSCT booklets

The same procedures for reviewing recommendations for refinement will be followed as described for Phase 1 Patients, CGs, Dyads and Clinicians.

#### Data analysis Phase 2

The same procedures for data analysis will be followed as described for Phase 1 HSCT Patients, CGs, Dyads and Clinicians.

## Discussion/significance

The impact of HSCT is usually viewed through the lens of the patient who has been diagnosed, has undergone treatment and HSCT, and is in remission. Accordingly, distress and burden experienced by patients throughout all HSCT phases is well established in the literature. The shift of HSCTs to the outpatient setting mandates CGs to participate in the illness experience throughout the course. The literature has established CGs as vulnerable and in need of coping interventions designed to enable them to continue providing care during an unpredictable recovery trajectory that may endure up to 5 years [39]. However, current interventions are few, and often lack in addressing CGs’ needs.

Studies suggest that optimal HSCT interventions should be dyadic; tailored to their specific needs; flexible in timing, content and format; and administered in a controlled manner to minimize information overload for the dyad [20, 24, 40]. Such interventions would ideally promote adaptive coping skills and stress management during the first 100 days post-HSCT, while laying the groundwork for effective, long-term psychosocial adaptation [10, 41, 42]. Interventions targeted towards adaptive coping strategies for dyads are likely to improve both CG-delivered care to the patient and the psychological and physical well-being of the CG.

Expanding upon this body of knowledge, this study is an initial step toward generating an evidence-informed, accessible, sustainable intervention that is adapted to facilitate coping of HSCT patients, CGs and dyads throughout the post-HSCT recovery phases. Adapting the CT booklets will expeditiously generate a supportive care intervention tailored to their needs. Findings will contribute further to the understanding of illness-related experiences while providing evidence for clinicians to better support patients and CGs throughout the recovery trajectory. Additionally, this study will inform subsequent intervention work through reliance on a systematic adaptation method.

There are some limitations to this study. First, data collection will take place at one clinical site in the US. It is likely that the experiences of HSCT patients, CGs and dyads at other HSCT centers are different, partially owing to the geographical location. The unique metropolitan and cultural characteristics of the study site can impact the generalizability of the findings to other areas, including rural and international. Considering the paucity of reports, it is anticipated that the findings from this study will add to the existing literature. Second, the interview questions are designed to elicit participants’ experiences with the unmodified CT and HSCT-adapted CT manuals. The accuracy of information relies on their truthful descriptions. Social desirability to impress and please the interviewer can pose a potential limitation. Despite explanations in the consent forms as well as verbal reminders that there is no right or wrong answer, some participants may still repress or embellish information. Third, there is no systematic process for integrating divergent recommendations from HSCT participants and clinicians. One of the challenges relates to differences in patients’ health beliefs and physicians’ perceptions of patients’ health beliefs. Last, since the manuals are written in English, the cultural relevance for non-native English-speaking participants is limited.

## Abbreviations

CBI: Couple-based Intervention
CDC: Centers for Disease Control and Prevention
CG: Caregiver
CT: *Coping Together*
HSCT CT: HSCT-adapted *Coping Together*
HSCT: Hematopoietic Stem Cell Transplantation
IRB: Institutional Review Board
MD: Medical Doctor
MSKCC: Memorial Sloan-Kettering Cancer Center
NP: Nurse Practitioner
QCA: Qualitative Content Analysis
RCT: Randomized control trial
RN: Registered Nurse
SW: Social Worker
